# USP10 inhibits the dopamine-induced reactive oxygen species–dependent apoptosis of neuronal cells by stimulating the antioxidant Nrf2 activity

**DOI:** 10.1016/j.jbc.2021.101448

**Published:** 2021-11-24

**Authors:** Junya Sango, Taichi Kakihana, Masahiko Takahashi, Yoshinori Katsuragi, Sergei Anisimov, Masaaki Komatsu, Masahiro Fujii

**Affiliations:** 1 Division of Virology, Niigata University Graduate School of Medical and Dental Sciences, Niigata, Japan; 2 Department of Physiology, Juntendo University Graduate School of Medicine, Bunkyo-ku, Japan

**Keywords:** Parkinson's disease, USP10, dopamine, ROS, Nrf2, p62, Keap1, apoptosis, PD, Parkinson's disease, DMEM, Dulbecco's modified Eagle's medium, FBS, fetal bovine serum, HO-1, heme oxygenase-1, HRP, horseradish peroxidase, NQO1, NAD(P)H dehydrogenase quinone 1, Nrf2, nuclear factor erythroid 2–related factor, ROS, reactive oxygen species, RPS, ribosomal protein, SDS, sodium dodecyl sulfate, siRNA, small interfering RNA, USP10, ubiquitin-specific protease 10

## Abstract

Nrf2 is an antioxidant transcriptional activator in many types of cells, and its dysfunction plays key roles in a variety of human disorders, including Parkinson's disease (PD). PD is characterized by the selective loss of dopaminergic neurons in PD-affected brain regions. Dopamine treatment of neuronal cells stimulates the production of reactive oxygen species (ROS) and increases ROS-dependent neuronal apoptosis. In this study, we found that the ubiquitin-specific protease 10 (USP10) protein reduces dopamine-induced ROS production of neuronal cells and ROS-dependent apoptosis by stimulating the antioxidant activity of Nrf2. USP10 interacted with the Nrf2 activator p62, increased the phosphorylation of p62, increased the interaction of p62 with the Nrf2 inhibitor Keap1, and stimulated Nrf2 antioxidant transcriptional activity. In addition, USP10 augmented dopamine-induced Nrf2 translation. Taken together, these results indicate that USP10 is a key regulator of Nrf2 antioxidant activity in neuronal cells and suggest that USP10 activators are promising therapeutic agents for oxidative stress–related diseases, including PD.

Reactive oxygen species (ROS) play a physiological and pathological role in various types of cells and tissues (1). For instance, intracellular ROS function as signal transduction molecules in various biological processes. However, their increased production has been implicated in many pathological conditions, such as neurodegenerative diseases, cardiovascular diseases, and cancer (2). Therefore, the amount of intracellular ROS is strictly regulated by several antioxidant systems.

Nuclear factor erythroid 2–related factor 2 (Nrf2) is an antioxidant factor that stimulates the transcription of a number of antioxidant genes, such as NAD(P)H dehydrogenase quinone 1 (NQO1) and heme oxygenase-1 (HO-1), under basal and various oxidative stress conditions (3, 4). Nrf2 activity is negatively and positively regulated by Kelch-like ECH-associated protein (Keap1) and p62 (SQSTM1), respectively. Prior to oxidative stress, the level of Nrf2 protein is low, since Nrf2 is ubiquitinated and continuously degraded by the proteasome (5). Nrf2 ubiquitination is regulated by interaction with Keap1, which acts as an Nrf2 adaptor to the ubiquitin ligase complex containing Cullin-3. However, during oxidative stress, three mechanisms enhance Nrf2 activity. First, oxidants induce cysteine modification of Keap1, which decreases Keap1's affinity for Nrf2 (5). Second, an oxidant induces the phosphorylation of serine 349 of p62 (pp62-S349), enhancing the interaction of p62 with Keap1 and releasing Nrf2 from Keap1 (6). Third, oxidants enhance the translation of Nrf2, which increases the amount of Nrf2 protein (7, 8, 9). Thus, Nrf2 activity is tightly regulated under both basal and oxidative stress conditions.

Dysfunction of the Nrf2/Keap1/p62 system has been associated with many neurodegenerative disorders, including Parkinson's disease (PD) (10). PD is characterized by the degeneration and death of dopaminergic neurons, which have cell bodies in the substantia nigra and axons projecting to the caudate nucleus and putamen (striatum) (11). While dopamine is a key neurotransmitter secreted by neurons that signal other neurons, accumulating evidence suggests that dopamine-induced ROS production plays a key role in the selective cell death of dopaminergic neurons in PD (12). For instance, dopamine treatment of neuronal cells stimulates ROS production and induces ROS-dependent apoptosis (13).

Ubiquitin-specific protease 10 (USP10) is a deubiquitinase ubiquitously expressed in many cell types, including neurons, and it augments antioxidant activity in cultured cells (14, 15). For instance, USP10 depletion in cultured nonneuronal cells treated with arsenite has been shown to increase ROS production and enhance ROS-induced apoptosis (16).

In the present study, we found that USP10 inhibits the dopamine-induced ROS production and ROS-dependent apoptosis in neuronal cells by activating Nrf2-mediated antioxidant gene expression.

## Results

### USP10 depletion increases dopamine-induced apoptosis of neuronal cells

Dopamine stimulation of neurons induces ROS production, which has been shown to play a key role in cell death of dopaminergic neurons in PD (17). USP10 has been shown to inhibit ROS production and ROS-dependent apoptosis in nonneuronal cells (16). We therefore investigated whether or not USP10 plays a role in dopamine-induced neuronal cell death.

**Figure 1:**
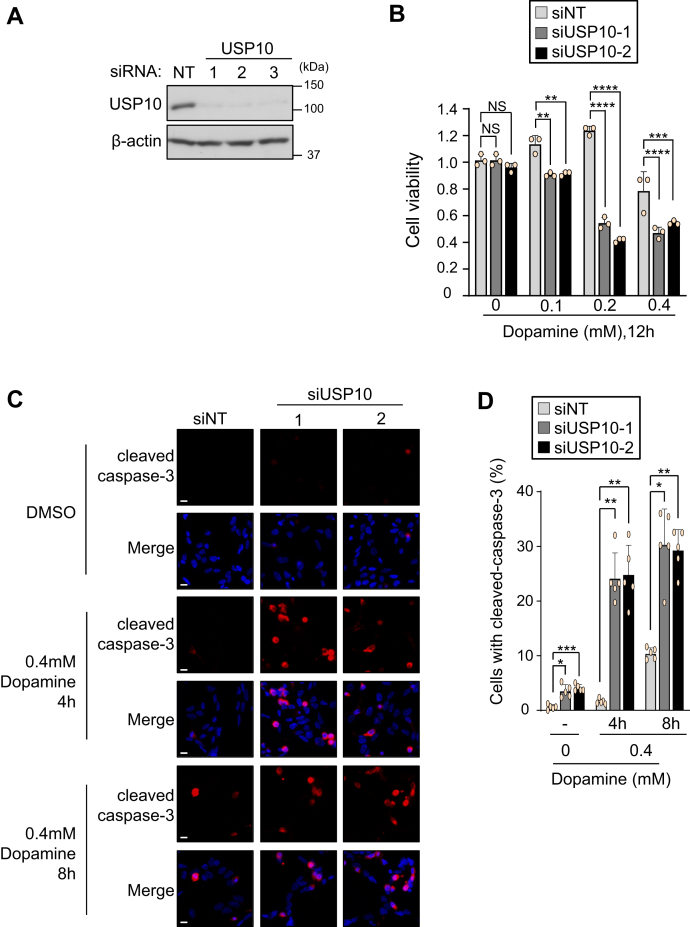
**USP10-KD enhances dopamine-induced apoptosis.***A*, SH-SY5Y cells were transfected with USP10-siRNA (USP10-1, 2, or 3) or control (NT) using Lipofectamine RNAiMAX. Whole cell lysates prepared from transfected cells were characterized by Western blotting with anti-USP10 and anti-β-actin antibodies. *B*, SH-SY5Y cells were transfected with USP10-siRNA (siUSP10-1 or 2) or control (siNT) using Lipofectamine RNAiMAX. The cells were then treated with 0.1 to 0.4 mM dopamine or DMSO for 12 h. The cells were treated with CCK-8 solution for 1 h. Culture supernatant was prepared from the transfected cells, and the absorbance (485 nm) of the culture supernatant was measured with an absorption meter (TriStar LB 941). The ratio of absorbance of cells to that of control siRNA (NT) treated with DMSO was presented as the mean and standard deviation (SD) of three samples. The significance of the differences was assessed by a one-way ANOVA, followed by Tukey's multiple comparison test. ∗∗*p* < 0.01; ∗∗∗*p* < 0.001; ∗∗∗∗*p* < 0.0001. *C* and *D*, USP10-KD (siUSP10-1 or 2) and control (siNT) cells were treated with 0.4 mM dopamine or DMSO for 4 and 8 h, and cells were then stained with anticleavage caspase-3 (*red*) and Hoechst 33258 (*blue*). The staining was evaluated with a fluorescence microscope. The ratio of cells with cleaved caspase-3 relative to total cells (measured by number of nuclei) was presented as the mean and SD of five samples in (*D*). The significance of the difference in Figure 1*D* was assessed by Brown–Forsythe and Welch ANOVA followed by Dunnett's T3 multiple comparisons test. ∗*p* < 0.05; ∗∗*p* < 0.01; ∗∗∗*p* < 0.001. Scale bar is 10 μm. ANOVA, analysis of variance; NS, Not significant; USP10, ubiquitin-specific protease 10.

SH-SY5Y is derived from neuroblastoma and has a phenotype similar to that of dopaminergic neurons (18). We reduced the expression of USP10 protein of SH-SY5Y using small interfering RNA (siRNA) targeting USP10 (siUSP10, USP10-KD). Western blotting detected a reduced expression of USP10 protein in USP10-KD cells (Fig. 1*A*). These USP10-KD cells were treated with 0.1 to 0.4 mM dopamine for 12 h, and then their cell viability was analyzed by measuring the cellular dehydrogenase activity. Dopamine treatment dose-dependently reduced the viability of USP10-KD cells, and the reduction in the number of cells was greater than that in USP10-WT cells (Fig. 1*B*).

Cleaved caspase-3 is a marker of apoptosis. Anticleaved caspase-3 staining detected apoptosis of USP10-KD cells treated with dopamine for 4 and 8 h, and the degree was greater than that in the control cells (siNT) (Fig. 1, *C* and *D*). These results showed that USP10 suppresses dopamine-induced apoptosis of SH-SY5Y cells.

### Depletion of USP10 increases the production of ROS

**Figure 2:**
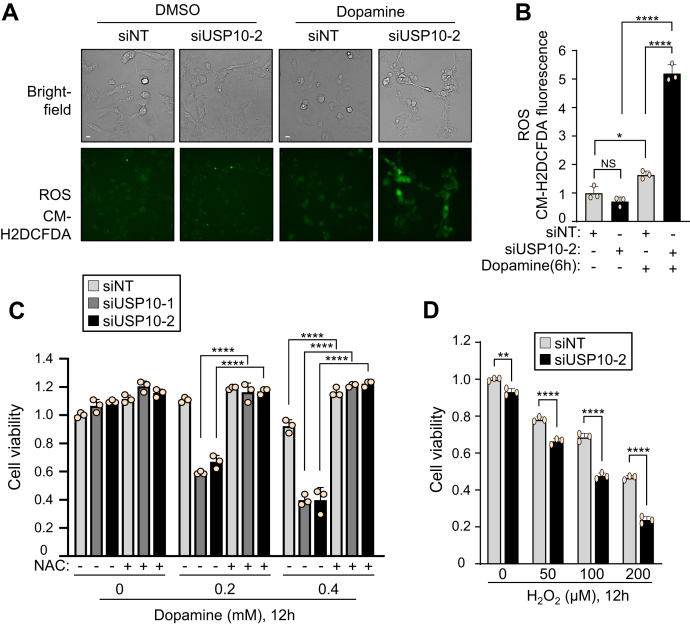
**USP10-KD augments dopamine-induced ROS production and ROS-dependent cell death.***A* and *B*, SH-SY5Y cells were transfected with USP10-siRNA (siUSP10-2) or control (siNT) using Lipofectamine RNAiMAX. Cells were treated with 5 μM CM-H2DCFDA (*Green*), an ROS-sensitive fluorescence dye, for 30 min. Transfected cells were then treated with 0.4 mM dopamine or DMSO for 6 h. The fluorescence intensity was measured. The ratio of fluorescence intensity of cells relative to that of the control (siNT) treated with DMSO was presented as the mean and SD from three samples. ∗*p* < 0.05; ∗∗∗∗*p* < 0.0001. Scale bar is 10 μm. Bright-field observations of cells were also presented. *C*, SH-SY5Y cells were transfected with USP10-siRNA (siUSP10-1 or 2) or control (siNT) using Lipofectamine RNAiMAX. Cells were pretreated with 500 μM N-acetyl cysteine 30 min before dopamine treatment and further treated with 0.2 to 0.4 mM dopamine or DMSO for 12 h. Cells were treated with CCK-8 solution for 1 h. Cell viability was evaluated by measuring the absorbance (485 nm) of culture medium with an absorbance meter (TriStar LB 941). The ratio of absorbance obtained from cells relative to that of the control (siNT) treated with DMSO was presented as the mean and SD from three samples. ∗∗∗∗*p* < 0.0001. *D*, USP10-KD (siUSP10-2) and control (siNT) cells were treated with 50 to 200 μM H_2_O_2_ for 12 h. Cell viability was measured with the CCK-8 kit. The absorbance obtained from cells was normalized to that of the control (siNT) treated with DMSO, and the ratio was presented as the mean and SD from three samples. ∗∗*p* < 0.01; ∗∗∗∗*p* < 0.0001. NS, not significant; USP10, ubiquitin-specific protease 10.

Dopamine-induced ROS production in neurons is a key factor in dopamine-induced apoptosis (2, 19). Therefore, we next investigated whether or not ROS play a role in dopamine-induced cell death of USP10-KD cells. ROS measurement using the ROS detector fluorescent probe CM-H2DCFDA showed that USP10-KD enhanced ROS production in SH-SY5Y cells (Fig. 2, *A* and *B*). Furthermore, the USP10-KD-enhanced cell death was attenuated by N-acetylcysteine (NAC), a precursor of the ROS scavenger glutathione (Fig. 2*C*). These results indicated that USP10-KD increases dopamine-induced cell death by enhancing the ROS production in SH-SY5Y cells.

Hydrogen peroxide (H_2_O_2_) is an endogenous ROS that is upregulated under many oxidative stress conditions. We investigated whether or not USP10 regulates H_2_O_2_-induced cytotoxicity in SH-SY5Y cells (Fig. 2*D*). H_2_O_2_ treatment for 12 h induced a dose-dependent decrease in the viability of SH-SY5Y cells (siNT), which was further enhanced by USP10-KD (siUSP10). These results showed that USP10 regulates cell death in SH-SY5Y cells under two distinct oxidative stress conditions.

### USP10 regulates the antioxidant Nrf2 activity in dopamine-treated neuronal cells

The expression of antioxidant genes mitigates cytotoxicity induced by various oxidative stresses. Nrf2 is a master antioxidant transcriptional activator that responds to oxidative stress and attenuates oxidative stress–induced cellular damage. In the basal state, Nrf2 is continuously ubiquitinated and degraded by the ubiquitin-proteasome system. Such Nrf2 ubiquitination is stimulated by its interaction with Keap1, an Nrf2 adaptor to the cullin-3 ubiquitin ligase complex. However, exposure to oxidants stimulates the release of Nrf2 from Keap1 mainly by two mechanisms. First, the oxidant modifies the cysteine residues of Keap1, which reduces the interaction affinity of Keap1 and Nrf2. Second, the oxidant activates the Keap1 inhibitor p62 by phosphorylating the serine residue of p62 at position 349 (pp62-S349) (6, 20). pp62-S349 then interacts strongly with Keap1 and stimulates the release of Nrf2 from Keap1 (6).

**Figure 3:**
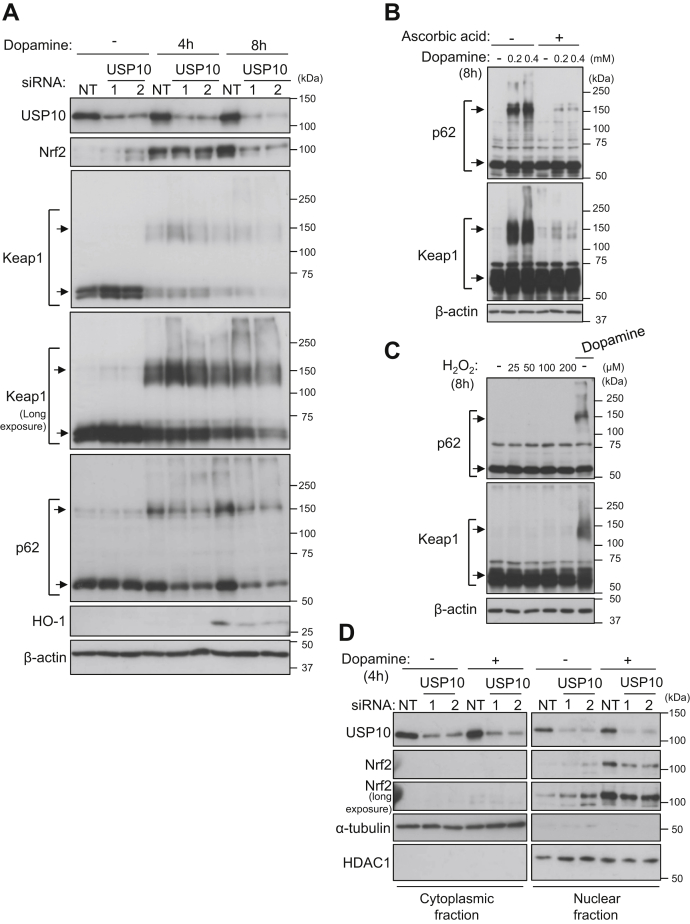
**USP10 regulates the antioxidant Nrf2/Keap1/p62 pathway in dopamine-treated neuronal cells.***A*, SH-SY5Y cells were transfected with USP10-siRNA (USP10-1 or 2) or control (NT) using Lipofectamine RNAiMAX. Cells were treated with 0.4 mM dopamine or DMSO for 4 and 8 h. Whole cell lysates prepared from transfected cells were characterized by Western blotting using the indicated antibodies. *B*, SH-SY5Y cells were pretreated with 0.3 mM ascorbic acid 30 min before dopamine treatment and then treated with 0.2 to 0.4 mM dopamine or DMSO for 8 h. Whole cell lysates prepared from these cells were characterized by Western blotting using the indicated antibodies. *C*, SH-SY5Y cells were treated with 25 to 200 μM H_2_O_2_ or 0.4 mM dopamine for 8 h. Whole cell lysates prepared from these cells were characterized by Western blotting using the indicated antibodies. *D*, SH-SY5Y cells were transfected with USP10-siRNA (USP10-1 or 2) or control (NT) using Lipofectamine RNAiMAX. Cells were treated with 0.4 mM dopamine or DMSO for 4 h. Lysates of nuclear and cytoplasmic fractions were prepared from transfected cells and characterized by Western blotting using the indicated antibodies. α-tubulin and HDAC1 were used as protein markers localized in the cytoplasm and nucleus, respectively. USP10, ubiquitin-specific protease 10.

Based on this information, we investigated whether or not USP10 regulates the Nrf2/Keap1/p62 antioxidant system in dopamine-induced ROS-dependent apoptosis in SH-SY5Y cells (Fig. 3). Dopamine treatment of USP10-WT cells (siNT) increased the levels of Nrf2 and the Nrf2-inducible gene HO-1, but the increases were reduced by USP10-KD (siUSP10) (Fig. 3*A*). In addition, dopamine treatment increased the amount of high-molecular-weight p62 bands (HMW-p62s) in USP10-WT cells, but the increase was attenuated by USP10-KD. Dopamine treatment of USP10-WT cells decreased the amount of Keap1 monomer and increased the amount of HMW-Keap1s. In contrast to Nrf2 and p62, USP10-KD did not decrease the amounts of Keap1 monomer or HMW-Keap1s with or without dopamine treatment, instead increasing the amount of Keap1 monomer without dopamine treatment (Fig. 3*A*). The molecular weights of HMW-p62s and HMW-Keap1s suggested that HMW-p62s and HMW-Keap1s were likely to be p62 multimers and Keap1 multimers, respectively. These findings suggested that the downregulation of Nrf2 and p62 and upregulation of Keap1 by USP10-KD were responsible for the increased dopamine-induced ROS production and ROS-dependent apoptosis of USP10-KD cells. It is noteworthy that unlike the dopamine-treated cells, the amount of Nrf2 in USP10-KD cells without dopamine treatment was higher than that in USP10-WT cells (Fig. 3). This result suggests that USP10 reduces the expression of Nrf2 in cells without dopamine treatment.

Next, we examined how dopamine induces HMW-p62 and HMW-Keap1 in SH-SY5Y cells. Ascorbic acid is a water-soluble antioxidant. Treatment with ascorbic acid decreased the amounts of HMW-p62 and HMW-Keap1 in dopamine-treated SH-SY5Y cells (Fig. 3*B*). Thus, the oxidative activity of dopamine is required for the induction of HMW-p62 and HMW in dopamine-treated cells. However, in contrast to dopamine, H_2_O_2_ treatment did not induce HMW-p62 and HMW-Keap1 in SH-SY5Y cells (Fig. 3*C*). Taken together, these results suggested that dopamine induces HMW-p62 and HMW-Keap1 in an oxidant-dependent and dopamine-specific mechanism.

Oxidative stress induces the translocation of Nrf2 from the cytoplasm to the nucleus, and nuclear Nrf2 activates the transcription of antioxidant genes. Four-hour dopamine treatment of USP10-WT cells increased the amount of nuclear Nrf2, but the increase was attenuated by USP10-KD (Fig. 3*D*). These results showed that USP10-KD reduced the amount of nuclear Nrf2 in dopamine-stimulated cells, thereby increasing ROS production.

### Depletion of either Nrf2 or p62 augments dopamine toxicity on neuronal cells

**Figure 4:**
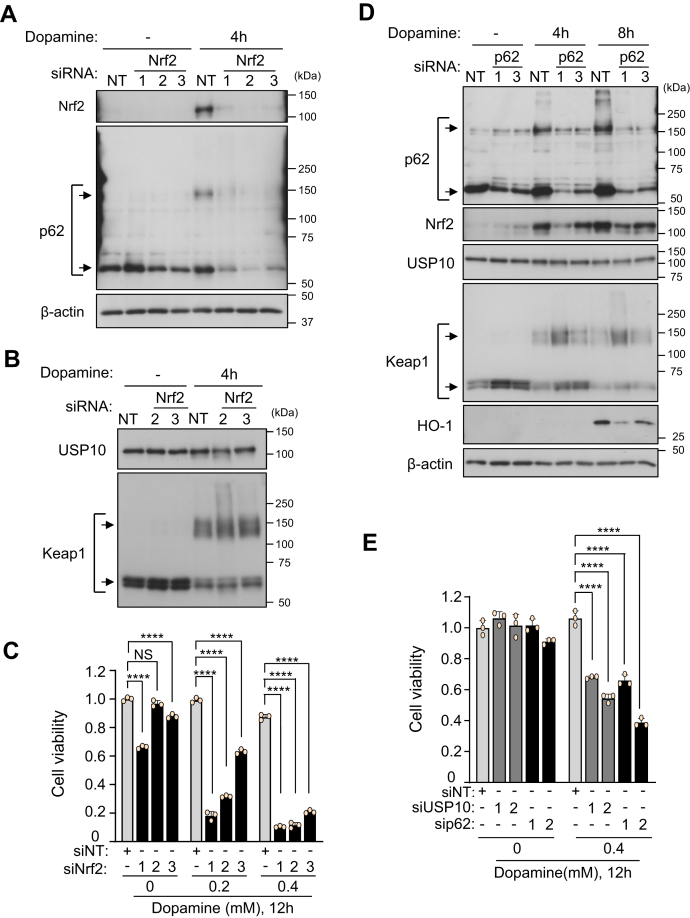
**Nrf2-KD and p62-KD enhance dopamine-induced cell death.***A* and *B*, SH-SY5Y cells were transfected with Nrf2-siRNA (Nrf2-1, 2, or 3) or control (NT) by Lipofectamine RNAiMAX. Cells were treated with 0.4 mM dopamine or DMSO for 4 h. Whole cell lysates prepared from transfected cells were characterized by Western blotting using the indicated antibodies. *C*, SH-SY5Y cells were transfected with Nrf2-siRNA (siNrf2-1, 2 or 3) or control (siNT) by Lipofectamine RNAiMAX. SH-SY5Y cells were treated with 0.2 to 0.4 mM dopamine or DMSO for 12 h. Cells were treated with CCK-8 solution for 1 h. Culture medium was prepared from transfected cells, and the absorbance (485 nm) of culture medium was measured by an absorbance meter (TriStar LB 941). The ratio of absorbance of the cells relative to that of the control siRNA (siNT) treated with DMSO was presented as the mean and SD from three samples. ∗∗∗∗*p* < 0.0001. NS, not significant. *D*, SH-SY5Y cells were transfected with p62-siRNA (p62-1 or 3) or control (NT) using Lipofectamine RNAiMAX. Cells were then treated with 0.4 mM dopamine or DMSO for 4 h or 8 h. Whole cell lysates prepared from transfected cells were characterized by Western blotting using the indicated antibodies. *E*, SH-SY5Y cells were transfected with p62-siRNA (sip62-1 or 2), USP10-siRNA (siUSP10-1 or 2) and the control (siNT) using Lipofectamine RNAiMAX. Cells were then treated with 0.4 mM dopamine or DMSO for 12 h and with CCK-8 solution for 1 h, and the cell viability was measured with the CCK-8 kit by measuring the absorbance (485 nm) of culture supernatant. The ratio of the absorbance of cells relative to that of the control (siNT) treated with DMSO was presented as the mean and SD from three samples. ∗∗∗∗*p* < 0.0001.

After dopamine treatment, USP10-KD in SH-SY5Y cells decreased the amounts of Nrf2 and p62 proteins (Fig. 3) and simultaneously enhanced dopamine-induced apoptosis (Fig. 1). Given that Nrf2 and p62 inhibit oxidant-induced cell death of various cell types, we next examined whether or not Nrf2 and p62 regulate the dopamine toxicity of SH-SY5Y cells. Nrf2-KD significantly enhanced the dopamine-induced SH-SY5Y cell death (Fig. 4, *A*–*C*). Western blotting showed that Nrf2-KD reduced the amount of p62 in dopamine-treated cells (Fig. 4*A*). These results are consistent with the fact that Nrf2 stimulates p62 transcription (4). In contrast, Nrf2-KD had little effect on the amount of Keap1 and USP10 in dopamine-treated cells (Fig. 4*B*).

Like Nrf2-KD, p62-KD enhanced cell death and the enhancement was comparable to USP10-KD (Fig. 4, *D* and *E*). p62-KD decreased the amount of Nrf2 and Nrf2-induced gene (HO-1) but increased the amount of Keap1 monomer. It should be noted that p62-KD had little effect on the amount of USP10, suggesting that USP10 is an upstream regulator of p62 in dopamine-induced apoptosis of SH-SY5Y cells. Taken together, these results support the finding that USP10 inhibits the ROS-dependent apoptosis of SH-SY5Y cells by promoting Nrf2 activity.

### Keap1-KD attenuates USP10-KD-augmentation of dopamine-induced cell death

**Figure 5:**
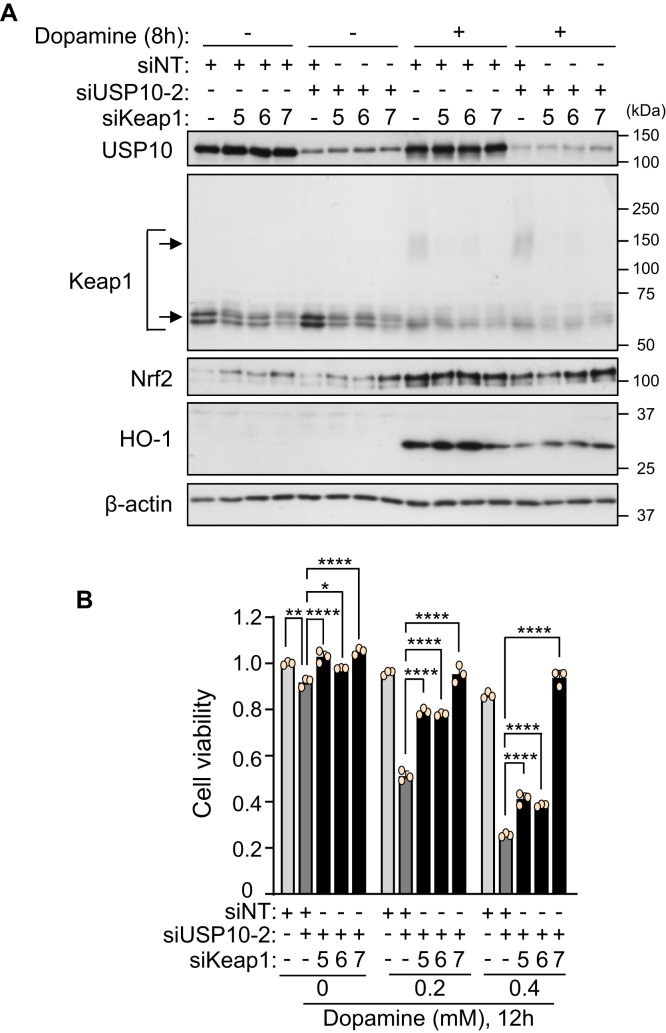
**Keap1-KD attenuates USP10-KD/dopamine-induced cell death.***A*, SH-SY5Y cells were transfected with USP10-siRNA (siUSP10-2), Keap1-siRNA (siKeap1-5, 6, or 7) or control (siNT) using Lipofectamine RNAiMAX. Cells were treated with 0.4 mM dopamine or DMSO for 8 h. Whole cell lysates were characterized by Western blotting using the indicated antibodies. *B*, SH-SY5Y cells were transfected with USP10-siRNA (siUSP10-2), Keap1-siRNA (siKeap1-5, 6 or 7), or control (siNT) using Lipofectamine RNAiMAX. Cells were then treated with 0.2 to 0.4 mM dopamine or DMSO for 12 h and with CCK-8 solution for 1 h, and the cell viability was measured with the CCK-8 kit by measuring the absorbance (485 nm) of the culture medium. The ratio of the absorbance of the cells to that of the control cells (siNT) treated with DMSO was presented as the mean and SD of three samples. ∗*p* < 0.05; ∗∗*p* < 0.01; ∗∗∗∗*p*  <  0.0001. USP10, ubiquitin-specific protease 10.

The data above indicated that USP10 attenuates dopamine-induced neuronal death by enhancing the antioxidant activity of Nrf2. In that case, USP10-KD-augmented dopamine-induced cell death should be reduced by inactivation of the Nrf2 inhibitor Keap1. To test this hypothesis, we reduced the Keap1 expression in USP10-KD cells (Fig. 5*A*). Western blotting showed that Keap1-KD reduced the amount of Keap1 with and without dopamine treatment (Fig. 5*A*). USP10-KD decreased the amount of Nrf2 and an Nrf2-inducible gene HO-1 in dopamine-treated cells, but the decreases were attenuated by Keap1-KD. Keap1-KD significantly reduced USP10-KD-enhanced dopamine toxicity against SH-SY5Y cells (Fig. 5*B*). Taken together, these results indicate that USP10 suppresses dopamine-induced SH-SY5Y cell death by stimulating the antioxidant activity of Nrf2, which is inhibited by Keap1.

### PERK, a kinase of Nrf2, is not involved in the reduction of antioxidant activity of Nrf2 by USP10-KD.

**Figure 6:**
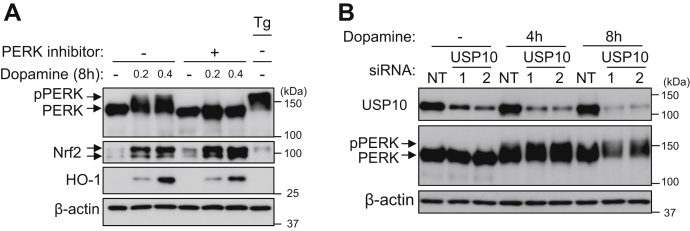
**Dopamine enhances PERK phosphorylation.***A*, SH-SY5Y cells were pretreated with 1 μM GSK260614 (PERK inhibitor) or DMSO 30 min before dopamine treatment and then treated with 0.2 to 0.4 mM dopamine or DMSO for 8 h. In addition, cells were treated with 1 μM thapsigargin (Tg) for 30 min, and this was used as positive control for PERK phosphorylation. Whole cell lysates prepared from these cells were characterized by Western blotting using the indicated antibodies. *B*, SH-SY5Y cells were transfected with USP10-siRNA (USP10-1 or 2) or control (NT) using Lipofectamine RNAiMAX. Cells were treated with 0.4 mM dopamine or DMSO for 4 and 8 h. Whole cell lysates prepared from transfected cells were characterized by Western blotting using the indicated antibodies. USP10, ubiquitin-specific protease 10.

PERK is a kinase that phosphorylates Nrf2 under various stress conditions (21, 22). The phosphorylation of Nrf2 by PERK enhances the antioxidant activity of Nrf2 by promoting its dissociation from Keap1 (21). We examined whether dopamine activates the kinase activity of PERK in SH-SY5Y cells by measuring the phosphorylation of PERK, a substrate of PERK (pPERK), by Western blotting (Fig. 6*A*). Dopamine treatment induced an upward shift in the mobility of some PERKs, and this shift was abolished by the PERK inhibitor GSK260414 (23). Thapsigargin, an activator of the kinase activity of PERK, also induced the upward migration of some PERKs in Western blotting. These results suggest that PERK with an upward mobility shift is phosphorylated PERK and that dopamine stimulates the autophosphorylation of PERK by PERK.

Dopamine treatment increased the amounts of Nrf2 and the Nrf2-induced gene HO-1, and the increase in HO-1, but not in Nrf2, was partially reduced by a PERK inhibitor, suggesting that PERK enhances dopamine-induced Nrf2 activity to HO-1. Next, we examined whether USP10 regulates the phosphorylation of PERK (pPERK) (Fig. 6*B*). USP10-KD slightly increased the amount of pPERK in SH-SY5Y cells treated with dopamine for 4 and 8 h. These results suggest that PERK is not involved in the Nrf2 activation by USP10 in dopamine-treated SH-SY5Y cells.

### USP10-KD does not reduce the amount of Nrf2 mRNA in neuronal cells

**Figure 7:**
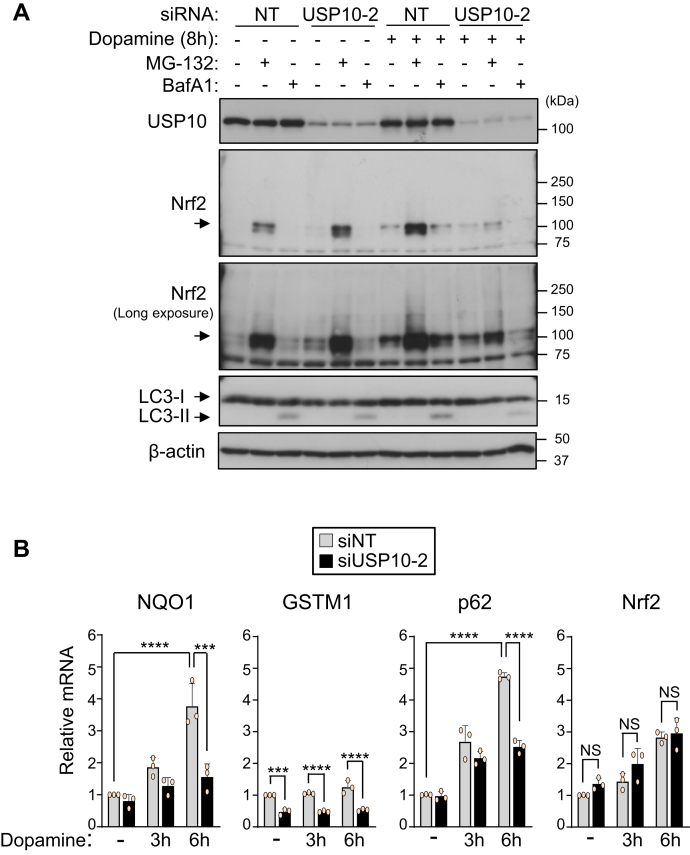
**USP10-KD does not reduce the amount of Nrf2 mRNA in neuronal cells.***A*, SH-SY5Y cells were transfected with USP10-siRNA (USP10-2) or control (NT) using Lipofectamine RNAiMAX. Cells were pretreated with 5 nM Bafilomycin A1 30 min before dopamine treatment and further treated with 0.4 mM dopamine or DMSO with or without 5 μM MG-132 for 8 h. Whole cell lysates prepared from transfected cells were characterized by Western blotting using the indicated antibodies. *B*, SH-SY5Y cells were transfected with USP10-siRNA (siUSP10-2) or control (siNT) using Lipofectamine RNAiMAX. Cells were treated with 0.4 mM dopamine or DMSO for 3 or 6 h. Total RNA was extracted from these cells, and the relative amount of NQO1, GSTM1, p62, and Nrf2 mRNA to β-actin mRNA was measured by real-time RT-PCR. The data were presented as the means ± SD (n = 3). ∗∗∗*p* < 0.001; ∗∗∗∗*p* < 0.0001. NS, Not significant; USP10, ubiquitin-specific protease 10.

The amount of Nrf2 protein is mainly regulated by proteasome-mediated degradation. To investigate how USP10-KD decreases the Nrf2 protein level in dopamine-treated cells, USP10-KD cells were treated with either a proteasome inhibitor (MG-132) or an autophagy inhibitor (BafA1) (Fig. 7*A*). Treatment with MG-132 but not BafA1 significantly increased the Nrf2 level in USP10-WT cells with and without dopamine treatment, suggesting that the Nrf2 protein is degraded by proteasome with and without dopamine treatment. MG-132 significantly increased the amount of Nrf2 in USP10-KD cells without dopamine treatment, but the increase with dopamine treatment was small and much less than that in USP10-WT cells. These results suggest that the degradation of Nrf2 protein by proteasome only partially explains the reduction in Nrf2 in dopamine-treated USP10-KD cells.

To understand the mechanism by which USP10 increases the amount of Nrf2 protein without inhibiting its degradation, we examined whether or not USP10-KD regulates the mRNA levels of Nrf2 and three Nrf2 target genes, NQO1, GSTM1, and p62. Dopamine treatment increased the levels of two Nrf2 inducible mRNAs (NQO1 and p62) in USP10-WT cells, but their levels were decreased by USP10-KD (Fig. 7*B*). Regarding GSTM1, USP10-KD decreased the mRNA level of GSTM1 with and without dopamine treatment, suggesting that USP10 stimulates Nrf2 activity even without dopamine treatment. These results indicated that USP10-KD reduces dopamine-stimulated Nrf2 transcriptional activity. Importantly, in contrast to the other genes tested, the Nrf2 mRNA level was not reduced by USP10-KD, even though dopamine increased the level of Nrf2 mRNA. Given that the proteasome inhibitor MG-132 only partially reversed the decrease in Nrf2 protein in dopamine-treated USP10-KD cells (Fig. 7*A*), these results suggest that USP10-KD reduces the translation of Nrf2 in dopamine-treated cells.

### USP10-KD suppresses global translation in neuronal cells

**Figure 8:**
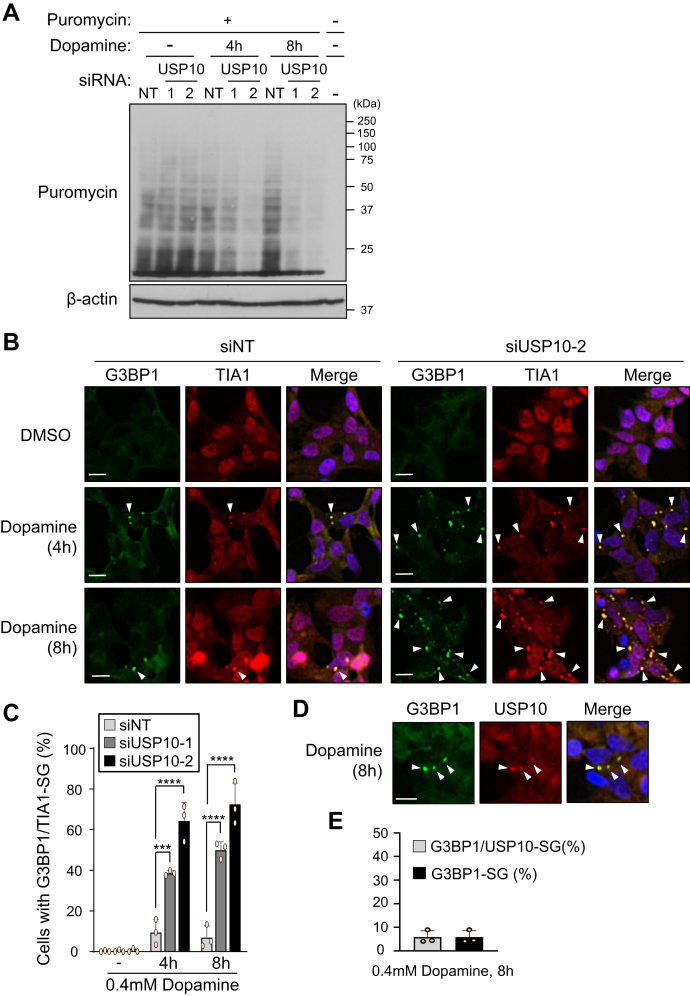
**USP10-KD suppresses global translation in dopamine-treated cells.***A*, SH-SY5Y cells were transfected with USP10-siRNA (USP10-1 or 2) or control (NT) using Lipofectamine RNAiMAX. Cells were treated with 0.4 mM dopamine or DMSO for 4 or 8 h and further treated with 10 μg/ml puromycin for 10 min. Cell lysates were characterized by Western blotting using the indicated antibodies. *B* and *C*, SH-SY5Y cells were transfected with USP10-siRNA (siUSP10-1 or 2) or control (siNT) using Lipofectamine RNAiMAX. Cells were treated with 0.4 mM dopamine or DMSO for 4 or 8 h and then stained with anti-G3BP1 (*green*) and anti-TIA1 (*red*) antibody. Nuclei were counterstained using Hoechst 33258 (*blue*). The proportions of cells containing G3BP1/TIA1-SG were presented as the mean and SD from three samples in (*C*). ∗∗∗*p* < 0.01; ∗∗∗∗*p* < 0.0001. *Arrowheads* indicate cells with G3BP1/TIA1-double-positive SG. Scale bar is 10 μm. *D* and *E*, SH-SY5Y cells were treated with 0.4 mM dopamine for 8 h and then stained with anti-G3BP1 (*green*) and anti-USP10 (*red*) antibodies. Nuclei were counterstained using Hoechst 33258 (*blue*). Scale bar is 10 μm. The proportions of cells containing G3BP1/USP10-SG or G3BP-1-SG were presented as the mean and SD from three samples in (*E*). USP10, ubiquitin-specific protease 10.

The data above suggested that USP10-KD decreases the dopamine-induced translation of Nrf2 in SH-SY5Y cells (Figs. 3 and 7). Next, we examined whether or not USP10 affects global translation in SH-SY5Y cells using a puromycin incorporation assay. Puromycin is incorporated into newly synthesized proteins, and its incorporation (new protein synthesis) is detected by antipuromycin antibody. SH-SY5Y cells were treated with dopamine and then further treated with puromycin for 10 min. Cell lysates were characterized by antipuromycin antibody. USP10-KD slightly increased new protein synthesis without dopamine treatment but significantly repressed protein synthesis with dopamine treatment (Fig. 8*A*). These results indicated that USP10-KD suppresses global translation in dopamine-treated SH-SY5Y cells.

Under various stress conditions including oxidative stress, protein synthesis (translation) from many mRNAs is repressed, reducing the amount of misfolded proteins and protecting cells from the toxicity of misfolded proteins, thereby promoting recovery from stress. Stress granules (SGs) are a key mechanism associated with translational repression under various stress conditions (24). SGs are stress-inducible cytoplasmic RNA granules that contain many translationally inactivated mRNAs and translation-initiating factors (25). USP10 is localized in SGs under various stress conditions and positively or negatively regulates SG formation, depending on the type of stress and cell (16, 26). To understand the role of USP10 in Nrf2 translation, we examined the SG formation in USP10-KD cells. G3BP1 and TIA1 are markers of SG. Dopamine treatment induced SG formation in USP10-KD cells, and the level was much higher than that in USP10-WT cells (Fig. 8, *B* and *C*). USP10 was localized in G3BP1-SG in dopamine-treated USP10-WT cells (Fig. 8, *D* and *E*). This finding is consistent with the localization of USP10 in G3BP1-SG in nonneuronal cells under various stress conditions (16, 26). These results suggest that the increase in SG formation by USP10-KD in dopamine-treated cells is associated with the suppression of global translation in dopamine-treated USP10-KD cells.

**Figure 9:**
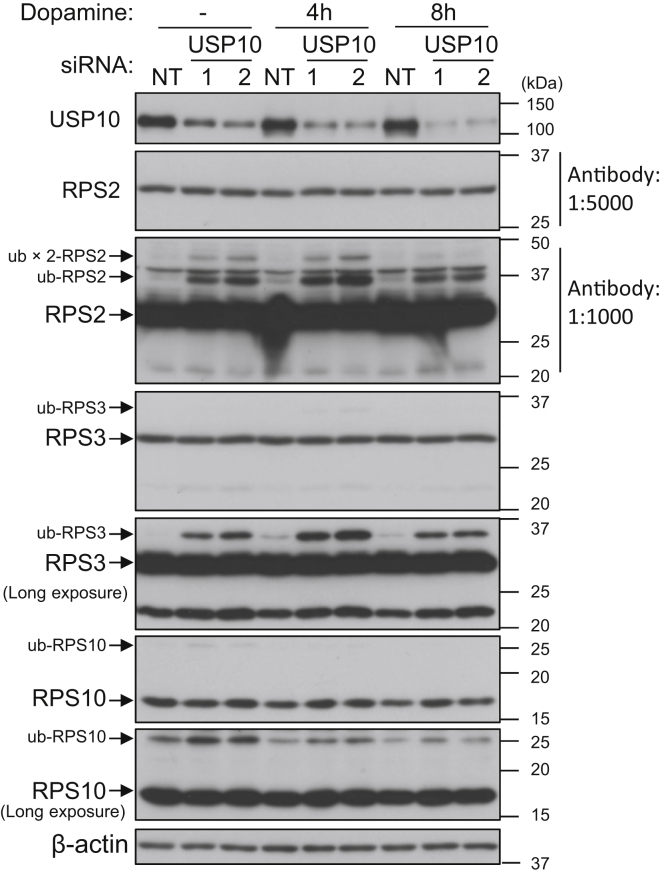
**USP10-KD induces ubiquitination of ribosomal proteins.** SH-SY5Y cells were transfected with USP10-siRNA (USP10-1 or 2) or control (NT) using Lipofectamine RNAiMAX. Cells were treated with 0.4 mM dopamine or DMSO for 4 and 8 h. Whole cell lysates prepared from transfected cells were characterized by Western blotting using the indicated antibodies. The experiments in Figures 6*B* and 9 were performed simultaneously as one experiment. Therefore, the data for β-actin and USP10 shown in Figure 6*B* are also represented in Figure 9. USP10, ubiquitin-specific protease 10.

Ribosomes are a component of the translation machinery. The repression of translation by various stresses is accompanied by the ubiquitination of several ribosomal proteins. USP10 is known to deubiquitinate ribosomal proteins, such as ribosomal protein S2 (RPS2), ribosomal protein S3 (RPS3), and ribosomal protein S10 (RPS10), and inhibit their degradation in lysosomes (27). Therefore, we examined whether USP10 regulates the ubiquitination of RPS2, RPS3, and RPS10 in SH-SY5Y cells treated with dopamine (Fig. 9). The amounts of ubiquitinated-RPS2 (ub-RSP2), ub-RPS3, and ub-RPS10 were increased in USP10-KD cells before dopamine treatment, while the amounts of nonubiquitinated-RPS2/RPS3/RPS10 were unchanged. Treatment with dopamine for 4 h in both USP10-KD and USP10-WT cells increased the amount of ub-RPS2 and ub-RPS3. In addition, the treatment from 4 to 8 h decreased the amount of ub-RPS2 and ub-RPS3. On the other hand, the amount of ub-RPS10 was decreased by dopamine treatment. These results suggest that increased ub-RPS2, ub-RPS3, and/or ub-RPS10 levels may be involved in the repression of translation in dopamine-treated USP10-KD cells.

### USP10-KD reduces the amount of phosphorylated p62

**Figure 10:**
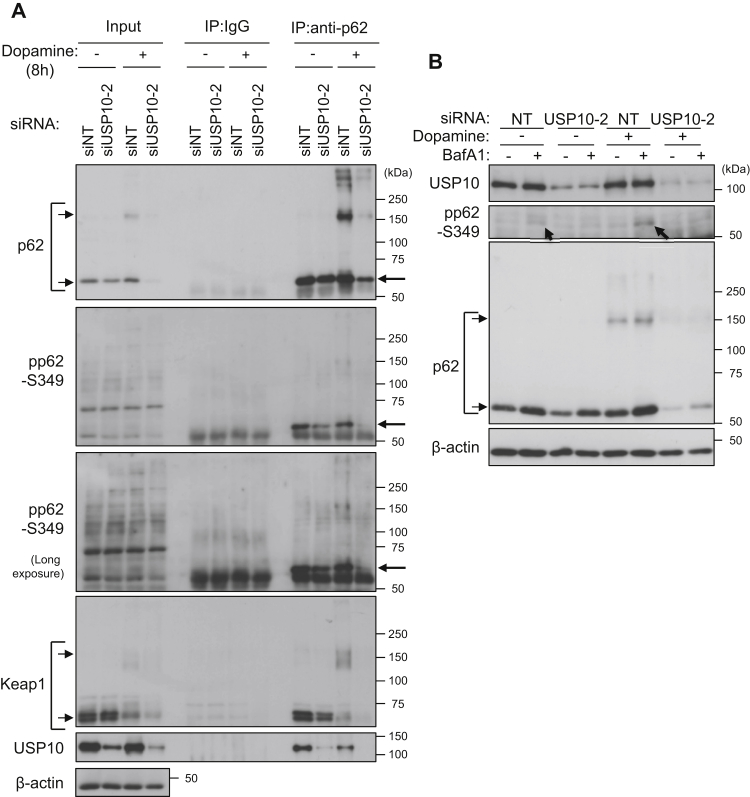
**USP10-KD reduces the amount of phosphorylated p62.***A*, SH-SY5Y cells were transfected with USP10-siRNA (siUSP10-2) or control (siNT) using Lipofectamine RNAiMAX. Cells were treated with 0.4 mM dopamine or DMSO for 8 h. Cell lysates prepared from transfected cells were immunoprecipitated with anti-p62 antibody or normal rabbit IgG. Cell lysates (input) and immunoprecipitates (IP) were characterized by Western blotting using the indicated antibodies. *B*, SH-SY5Y cells were transfected with USP10-siRNA (siUSP10-2) or control (siNT) using Lipofectamine RNAiMAX. The cells were pretreated with 5 nM BafA1 30 min before dopamine treatment and further treated with 0.4 mM dopamine or DMSO for 8 h. Cell lysates were characterized by Western blotting using the indicated antibodies. USP10, ubiquitin-specific protease 10.

Phosphorylated p62 at serine 349 (pp62-S349) activates Nrf2 much more strongly than unphosphorylated p62 (6). We therefore investigated whether or not USP10 affects the amount of pp62-S349 in SH-SY5Y cells (Fig. 10*A*). An immunoprecipitation analysis showed that USP10-KD with and without dopamine treatment decreased the amount of pp62-S349 and simultaneously reduced the interaction of p62 with Keap1. p62 interacted with USP10 in cells without dopamine treatment, and the interaction was slightly reduced by dopamine treatment. These results suggested that USP10 interacts with p62 to increase the amount of pp62-S349 and that pp62-S349 increased by USP10 reduces the degradation of Nrf2 in dopamine-treated cells.

pp62-S349 has been shown to be degraded by autophagy (28). In contrast to the immunoprecipitation analysis, Western blotting failed to detect pp62-S349 in SH-SY5Y with or without dopamine treatment, but pp62-S349 in SH-SY5Y cells was detected in BafA1-treated cells, and the amount was increased by dopamine treatment (Fig. 10*B*). In contrast to USP10-WT cells, little pp62-S349 was detected in BafA1/dopamine-treated USP10-KD cells. These results suggested that USP10-KD reduces the amount of pp62-S349, and the reduction is not mediated by autophagic degradation of pp62-S349.

### USP10-KD reduces p62-body formation in neuronal cells

**Figure 11:**
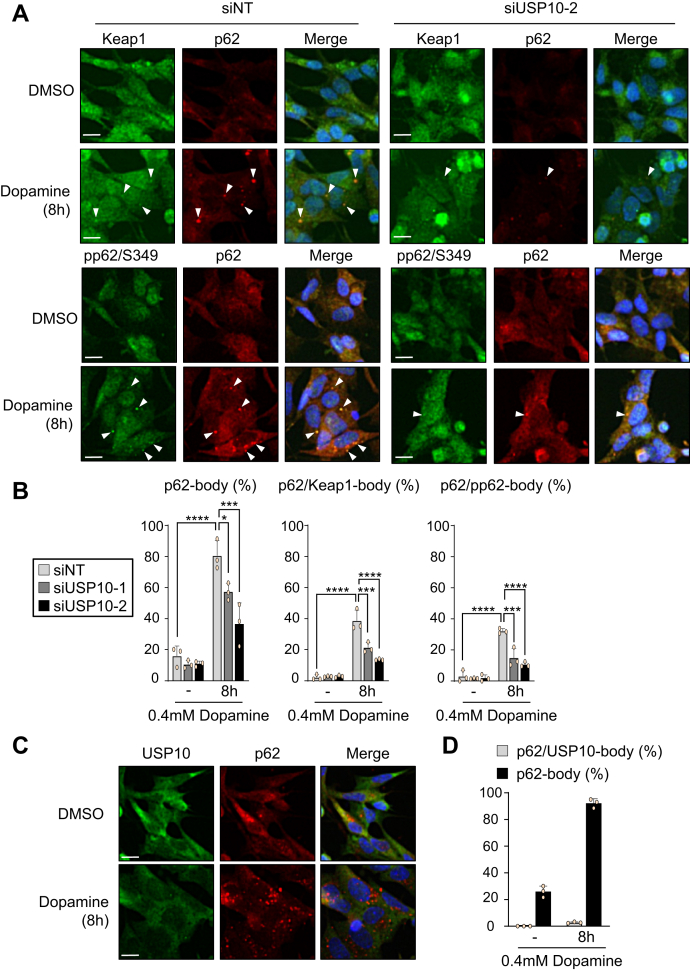
**USP10-KD reduces p62/Keap1-body formation in dopamine-treated cells.***A* and *B*, SH-SY5Y cells were transfected with USP10-siRNA (siUSP10-1 or 2) or control (siNT) using Lipofectamine RNAiMAX. Cells were treated with 0.4 mM dopamine or DMSO for 8 h and then stained with anti-Keap1 (*green*), anti-phospho-p62 (*green*), and anti-p62 (*red*) antibody. Nuclei were counterstained using Hoechst 33258 (*blue*). The proportions of cells containing p62-body, p62/Keap1-body, or p62/pp62-body were presented as the mean and SD from three samples in (*B*). ∗*p* < 0.05; ∗∗∗*p* < 0.001; ∗∗∗∗*p* < 0.0001. Scale bar is 10 μm. The *arrowheads* indicate cells with p62-body, p62/Keap1-body, or p62/pp62-body. *C* and *D*, SH-SY5Y cells were transfected with control siRNA using Lipofectamine RNAiMAX. Cells were treated with 0.4 mM dopamine or DMSO for 8 h and then stained with anti-USP10 (*green*) and anti-p62 (*red*) antibodies. Nuclei were counterstained using Hoechst 33258 (*blue*). Scale bar is 10 μm. The proportions of cells containing p62/USP10-body or p62-body were presented as the mean and SD from three samples in (*D*). USP10, ubiquitin-specific protease 10.

Oxidative stress induces p62 and pp62-S349 to form a protein condensation called a p62-body, and the p62-body colocalizes with Keap1 and induces degradation of both Keap1 and p62 (29, 30, 31). We therefore investigated whether or not USP10 controls the formation of p62-body (Fig. 11). Dopamine treatment induced the formation of p62-body, p62/Keap1-body, and p62/pp62-body in USP10-WT cells, but these condensate formations were reduced in USP10-KD cells (Fig. 11, *A* and *B*). These results suggest that USP10 increases the amount of p62/Keap1-body formation in dopamine-treated cells, thereby attenuating the Keap1 inhibitory activity of Nrf2. p62 interacted with USP10 in SH-SY5Y cells (Fig. 10*A*), but USP10 hardly colocalized with the p62-body in cells treated with dopamine (Fig. 11, *C* and *D*). These results suggested that protein–protein interaction between p62 and USP10 is not necessary for the formation of p62-bodies in dopamine-treated cells.

### Phosphorylated p62 attenuates the USP10-KD-augmentation of dopamine-induced cell death

**Figure 12:**
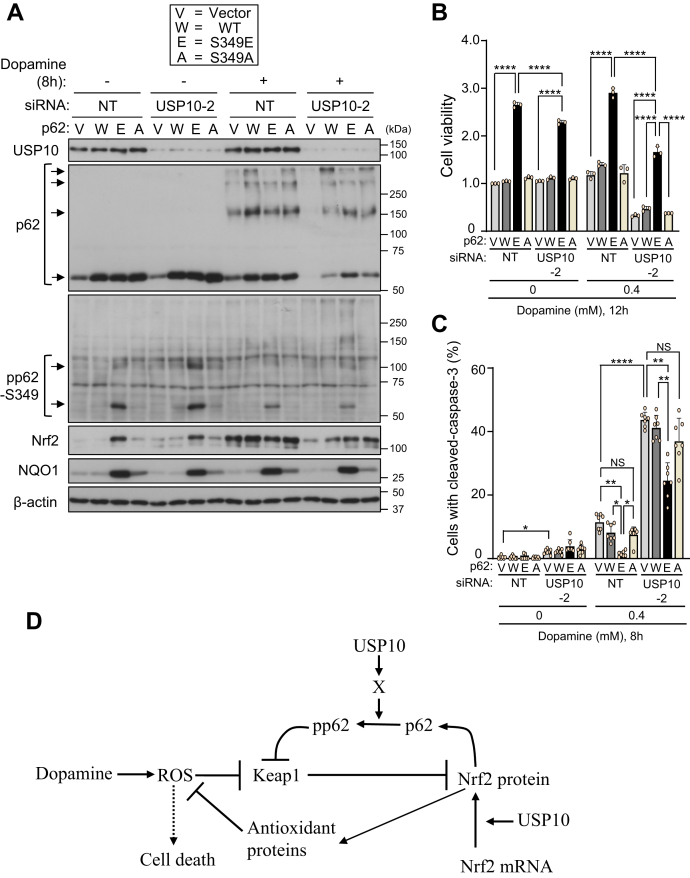
**Phosphorylation-mimicking p62 attenuated USP10-KD-induced cell death.***A*, SH-SY5Y cells stably expressing WT-p62 (W), p62-S349E (E) or p62-S349A (A) or the vector plasmid (V) were established. These SH-SY5Y cells were transfected with USP10-siRNA (USP10-2) or control (NT) by Lipofectamine RNAiMAX. Transfected cells were treated with 0.4 mM dopamine or DMSO for 8 h, and the whole cell lysates were characterized by Western blotting using indicated antibodies. *B*, the indicated SH-SY5Y cells were transfected with USP10-siRNA (USP10-2) or control (NT) by Lipofectamine RNAiMAX. Transfected cells were treated with 0.4 mM dopamine or DMSO for 12 h. Cells were treated with CCK-8 solution for 1 h, and culture medium was prepared from CCK-8-treated cells. The absorbance (485 nm) of culture medium was then measured with an absorbance meter (TriStar LB 941). The ratio of absorbance obtained from cells relative to that of the control cells (V) transfected with siNT (NT) treated with DMSO was presented as the mean and SD from three samples. ∗∗∗∗*p* < 0.0001. *C*, the indicated SH-SY5Y cells were transfected with USP10-siRNA (USP10-2) or control (NT) by Lipofectamine RNAiMAX. Transfected cells were treated with either 0.4 mM dopamine or DMSO for 8 h, and then the cells were stained with anti-cleaved caspase-3 and Hoechst 33258. The staining was evaluated by a fluorescence microscope. The ratio of cells with cleaved caspase-3 staining relative to total cells measured by the number of nuclei was presented as the mean and SD of seven samples in (*C*). The significance of the differences was assessed by Brown–Forsythe and Welch ANOVA followed by Dunnett's T3 multiple comparisons test. ∗*p* < 0.05; ∗∗*p* < 0.01; ∗∗∗∗*p* < 0.0001. *D*, the current model of USP10 regulation of Nrf2-dependent antioxidant activity in neuronal cells. USP10 activates the antioxidant activity of Nrf2 through two mechanisms. First, USP10 promotes phosphorylation of p62 (pp62-S349) *via* an unknown molecule (X), such as a phosphatase or kinase. Second, USP10 promotes the dopamine-induced translation of Nrf2 through inhibition of SG formation and/or deubiquitination of ribosomal proteins. NS, Not significant; USP10, ubiquitin-specific protease 10.

The reduced amount of pp62-S349 in USP10-KD cells was correlated with dopamine-induced cell death (Figs. 1 and 10). To assess the protective role of pp62-S349 in dopamine-induced cell death, we established SH-SY5Y cells that express a phosphorylation-mimic p62 mutant (p62-S349E, Ser to Glu mutation at position 349 of p62), phosphorylation-defective p62 mutant (p62-S349A, Ser to Ala mutation at position 349 of p62), or p62-WT. Anti-p62 antibody detected comparable amounts of p62-S349E, p62-S349A, and p62-WT in SH-SY5Y cells, and these levels were much higher than those of endogenous p62 in vector-transfected cells (Fig. 12*A*). A high amount of pp62-S349 was detected in p62-S349E-transfected cells, whereas only small amounts were detected in p62-WT or p62-S349A cells. p62-S349E increased the amount of Nrf2 equivalently in both USP10-KD and USP10-WT cells without dopamine treatment, and these increases were barely detectable in p62-WT and p62-S349A. Dopamine treatment increased the amount of Nrf2 in USP10-WT cells expressing p62-WT and p62-S349A, and the amount was comparable to that in USP10-WT cells expressing p62-S349E. The amount of Nrf2 in dopamine-treated USP10-KD cells expressing three p62 was less than that in USP10-WT cells. In addition, p62-S349E increased the amount of Nrf2-induced gene NQO1 in both USP10-KD and USP10-WT cells with or without dopamine treatment, and these increases were barely detectable in p62-WT and p62-S349A.

We next examined the activities of three p62 proteins to dopamine toxicity to SH-SY5Y cells. The overexpression of p62-S349E in dopamine-treated USP10-KD cells prominently increased cell viability and reduced apoptosis, but these effects were not observed by p62-S349A or p62-WT (Fig. 12, *B* and *C*). These results indicated that Nrf2 activation by p62-S349E in USP10-KD cells reduced dopamine toxicity to USP10-KD cells. It should be noted that the dopamine-induced toxicity to USP10-KD cells expressing p62-S349E was still higher than that of USP10-WT cells expressing p62-S349E (Fig. 12*B*). These results suggest that USP10 has a different activity from increasing p62 phosphorylation (pp62-S349) in suppressing dopamine-induced cell death.

## Discussion

The antioxidant activity of Nrf2/Keap1/p62 system plays an important protective role in various oxidative stresses in neurons. In the present study, we found that USP10 in neuronal cells reduces dopamine-induced ROS production and ROS-dependent apoptosis by stimulating antioxidant activity of Nrf2. These results indicate that USP10 is a key player in protecting neurons from oxidative stress–induced apoptosis by stimulating Nrf2 antioxidant activity. Dopamine has been reported to induce not only apoptosis but also cell death by autophagy in neuronal cells (32). Therefore, USP10 may be involved in not only apoptosis but also autophagic cell death in neurons.

We found that USP10 stimulates Nrf2 activity in neuronal cells through two mechanisms (Fig. 12*D*). First, USP10 promotes the dopamine-induced global translation including Nrf2 in SH-SY5Y cells (Figs. 7 and 8). In this regard, USP10 has been shown to deubiquitinate several ribosomal proteins and inhibit their lysosome-mediated degradation (27). We also found that USP10-KD increases the ubiquitination of RPS2, RPS3, and RPS10 in SH-SY5Y cells treated with and without dopamine. Therefore, USP10-KD-induced ribosome dysfunction may inhibit global translation in dopamine-treated cells. Further analyses are needed to understand how USP10 controls the translation of Nrf2 under oxidative stress conditions.

As a second mechanism for activating Nrf2, USP10 increased the amount of pp62-S349 in neuronal cells, and this pp62-S349 then activated the Nrf2-dependent antioxidant activity (Figure 10, Figure 11, Figure 12). The increase in p62 phosphorylation by USP10 (pp62-S349) can be explained by several mechanisms. For instance, USP10 may stimulate p62 kinase or inhibit p62-phosphatase. Understanding the mechanism by which USP10 regulates Nrf2 activity is important for deepening our understanding of Nrf2-dependent antioxidant activity.

Unlike the dopamine-treated cells, the amount of Nrf2 in USP10-KD cells treated with dopamine was higher than that in USP10-WT cells (Figs. 3, *A* and *D* and 7*A*). This result suggests that USP10 reduces the expression of Nrf2 in cells without dopamine treatment. In the puromycin incorporation assay, USP10-KD slightly increased global protein synthesis in SH-SY5Y cells. Therefore, USP10 may decrease the translation of Nrf2 in SH-SY5Y cells without dopamine treatment

Accumulating evidence suggests that the dysfunction of Nrf2 and/or p62 promotes the development of PD (33, 34). α-Synuclein is a causative factor of familial and sporadic PD. Cytoplasmic α-synuclein-positive aggregates in PD neurons, called Lewy bodies, are the hallmark pathology of PD patients (35). Interestingly, USP10 and p62 have been shown to localize to Lewy bodies in PD neurons (14, 36). Furthermore, the coexpression of USP10 and α-synuclein in cultured cells treated with a proteasome inhibitor induces cytoplasmic α-synuclein/USP10/p62-positive aggregates that resemble Lewy bodies (14). These studies suggest that USP10 and p62 play a role in two characteristic pathologies in PD neurons: α-synuclein aggregation and inhibition of ROS-dependent neuronal apoptosis. It is important to clarify how these two activities of USP10 and p62 work together in the PD development process.

The expression of Nrf2 and USP10 in the brains of PD patients has been studied separately. According to an immunohistochemical study by Ramsey *et al*., Nrf2 is localized in the nuclei of neurons in the substantia nigra of PD patients, whereas it is expressed in the cytoplasm in normal human controls (37). These results suggest that high oxidative stress in PD patients induces the activation of Nrf2, but the activation is not sufficient to inhibit oxidative stress–induced neuronal toxicity. On the other hand, it has been reported that USP10 protein levels in the amygdala of PD patients are slightly increased compared with controls (14). Therefore, it will be important to clarify how USP10 regulates neuronal Nrf2 activity in the pathogenesis of PD.

The Nrf2/Keap1/p62 antioxidant system has been shown to play important roles in a variety of diseases, including neurodegenerative diseases and cancer, making it an attractive therapeutic target for the treatment of these diseases. USP10 was found to be a key regulator of the Nrf2/Keap1/p62 system and neuronal apoptosis. Thus, USP10 activity against the Nrf2/Keap1/p62 system is also a promising drug target for oxidative stress–associated diseases, including PD.

## Experimental procedures

### Cell lines and culture condition

SH-SY5Y is a human neuroblastoma cell line (18), and cells were cultured in Dulbecco's Modified Eagle's Medium (DMEM) (11965092; Thermo Fisher Scientific) supplemented with 5% heat-inactivated fetal bovine serum (FBS), 50% Opti-MEM (Thermo Fisher Scientific), 2 mM L-glutamine, 100 units/ml penicillin, 100 μg/ml streptomycin, and MEM Non-essential AA solution. To generate retroviral vectors expressing p62, we used the Platinum-E (Plat-E) retroviral packaging cell line expressing gag-pol and envelope proteins of Moloney murine leukemia virus, which were cultured in DMEM supplemented with 10% heat-inactivated FBS, 2 mM L-glutamine, 100 units/ml penicillin, 100 μg/ml streptomycin, and MEM Non-essential AA solution.

### Reagents and antibodies

The following reagents were purchased from the indicated companies: Dopamine hydrochloride (14212-71; Nacalai Tesque), MG-132 (474790; MERCK), bafilomycin A1 (B1793; Sigma-Aldrich), blasticidin (ant-bl-05; InvivoGen), N-acetylcysteine (A9165; Sigma-Aldrich), Puromycin dihydrochloride from *Streptomyces alboniger* (P8833; Sigma-Aldrich), Hydrogen peroxide solution (21676-3; MERCK), L(+)-ascorbic acid (012-04802, FUJIFILM Wako), GSK-2606414 (G7345; LKT Laboratories), Thapsigargin (T9033; MERCK) and Hoechst 33258 (H-3569; Thermo Fisher Scientific). The following antibodies were used in this study: anti-USP10 (HPA006731; Sigma-Aldrich), anti-p62 (PM045; MBL, GP62-C; PROGEN), anti-phospho-p62 (Ser351) antibody (PM074; MBL), anti-cleaved caspase-3 (9661S; Cell Signaling Technology), anti-Keap1 (10503-2-AP; Protein Tech), anti-Nrf2 (sc-13032; Santa Cruz Biotechnology), anti-heme oxygenase 1 (HO-1) (sc-136960; Santa Cruz Biotechnology), anti-LC3 (PM036B; MBL), anti-G3BP1 (611127; BD Transduction Laboratories), anti-TIA1 antibody (ab40693; Abcam), anti-NQO1 (3187; Cell Signaling Technology), anti-puromycin (clone12D10, MABE343; Sigma-Aldrich), anti-PERK antibody (3192; Cell Signaling Technology), anti-RPS2 antibody (A303-794A-T; Bethyl Laboratories), anti-RPS3 antibody (A303-840A-T; Bethyl Laboratories), anti-RPS10 antibody [EPR8545] (ab151550; Abcam), anti-β-actin (sc-47778; Santa Cruz Biotechnology), HDAC1 (05-614; Millipore), Normal rabbit IgG (PM035; MBL), and anti-α-tubulin (CP06; MERCK).

### RNA interference

Small interfering RNAs (siRNAs) specific to human USP10 RNA (Oligo ID: HSS113446, HSS113447), p62 RNA (Oligo ID: HSS113116, HSS113117), Nrf2 RNA (Oligo ID: HSS107130, HSS181505, HSS18506), and the negative control siRNA (Cat. No. 12935-100) were purchased from Thermo Fisher Scientific. siRNA specific to Keap1 RNA (Oligo ID: SI03246439, SI04288844, SI04267886, SI04155424) and the negative control siRNA (Cat. No. 1027280) were purchased from Qiagen. These siRNAs were transfected into cells using Lipofectamine RNAiMAX reagents according to the manufacturer’s protocol (Thermo Fisher Scientific).

### Plasmids

Retroviral expression plasmids for p62 and p62-mutants have been described previously (6).

### Western blotting

SH-SY5Y cells were lysed with sodium dodecyl sulfate (SDS) lysis buffer (125 mM Tris-HCl, pH 6.8, 4% SDS, 10% sucrose, 10% 2-mercaptoethanol, and 0.005% bromophenol blue). Nuclear and cytoplasmic fractionations were prepared using an NE-PER extraction kit (PI78833; Thermo Fisher Scientific). Cell lysates (approximately 10 μg of protein) were separated by SDS–polyacrylamide gel electrophoresis (PAGE), electrophoretically transferred onto a PVDF membrane (1620177; Bio-Rad). To suppress the nonspecific binding of antibodies to the membrane, PVDF membranes were treated with Blocking One (03953-95; Nacalai Tesque) or 5% skim milk dissolved in Tris Buffered Saline (137 mM NaCl, 2.7 mM KCl, 25 mM Tris-HCl, pH 7.4) with 0.2% Tween 20 for 30 min at room temperature. The membranes were then treated with the indicated primary antibodies diluted in Can Get Signal (NKB-101; TOYOBO) and further incubated with the secondary antibody of either mouse anti-rabbit IgG conjugated with horseradish peroxidase (HRP) (SC-2357; Santa Cruz) or goat anti-mouse IgG (heavy and light chain)-HRP (170-6516; Bio-Rad) diluted in Can Get Signal. Immunoreactive bands were detected with an enhanced chemiluminescence (ECL) detection system (ECL Western Blotting Detection Reagents [RPN2209; Cytiva) and visualized by Amersham Hyperfilm ECL films (28906837; Cytiva). To reduce nonspecific binding of phospho-p62 (ser351) antibody, PVDF membranes were treated with 5% skim milk dissolved in phosphate-buffered saline (PBS) (137 mM NaCl, 2.7 mM KCl, 8.1 mM Na2HPO4, 1.5 mM KH2PO4).

### The puromycin incorporation assay

To detect newly synthesized proteins, SH-SY5Y cells are incubated with puromycin at a final concentration of 10 μg/ml for 10 min before harvesting the cells. To detect puromycin incorporated into the proteins, cell lysates were characterized by Western blotting with antipuromycin antibody.

### Establishment of SH-SY5Y cells overexpressing p62 mutants

To establish a retrovirus expressing p62-WT or p62 mutant (p62-S349E, p62-S349A), Plat-E cells were transfected with the retrovirus vector expressing p62-WT or p62 mutant using FuGENE6, according to the manufacturer’s instructions. At 48 h after transfection, the culture supernatant containing viruses was collected and infected to SH-SY5Y cells in the presence of 8 μg/ml polybrene. At 24 h after infection, cells were cultured in medium containing 1 μg/ml puromycin. The expression of p62 or p62 mutants (p62-S349E and p62-S349A) in SH-SY5Y cells was measured by Western blotting.

### Immunofluorescent assay

SH-SY5Y cells were cultured on glass coverslips in 6-well plates and transfected with siRNA using Lipofectamine RNAiMAX reagent. Cells were fixed with 4% paraformaldehyde in PBS for 20 min, followed by permeabilization and blocking in blocking buffer (0.1% Triton X-100 and 0.2% gelatin dissolved in PBS) for 30 min. The cells were then treated with the primary antibodies diluted in blocking buffer for 60 min and further incubated with the secondary antibody of Alexa488-or Alexa594-conjugated goat anti-mouse, Alexa488-conjugated goat anti-rabbit, Alexa488- or Alexa594-conjugated donkey anti-rabbit, or Alexa594-conjugated goat anti-guinea pig antibody (A11029, A11032, A11034, A21206, A21207, A11076; Thermo Fisher Scientific) diluted in blocking buffer for 60 min. Cell nuclei were stained with Hoechst 33258. The samples were mounted in ProLong Glass Antifade Mountant (P36984; Thermo Fisher Scientific), and the stainings from three fields (>80 cells) were analyzed with a fluorescence microscope (BZ-X810; KEYENCE) and the FIJI ImageJ software program (https://imagej.net). p62-positive, p62/Keap1-positive, and pp62-S349/p62-positive condensates with a size >0.5 μm^2^ were then evaluated as p62-body, p62/Keap1-body, and p62/pp62-body, respectively. Cells containing more than two p62 bodies were evaluated as cells with a p62-body. The proportions (%) of cells with a p62/Keap1-body, p62-body, or p62/pp62-body relative to total cells were presented as the mean and SD. To measure SG formation, stainings of three fields (>80 cells) were analyzed by a microscope and the FIJI ImageJ software program, and G3BP1/TIA1-double-positive condensates with a size >0.25 μm^2^ were evaluated as SG. The proportions (%) of cells with SG relative to total cells were presented as the mean and SD

### Coimmunoprecipitation assay

SH-SY5Y cells were cultured in a 100-mm-dish (430167; Corning) and transfected with siRNA (USP10-siRNA) using Lipofectamine RNAiMAX reagent. Cells were lysed with ice-cold NP-40 lysis buffer (1% Nonidet P-40, 25 mM Tris-HCl, pH 7.4, 150 mM NaCl, 1 mM EDTA, 1 mM phenylmethylsulfonyl fluoride, 20 μg/ml aprotinin), and lysates were precleaned by protein G-sepharose (17-0618-01; Cytiva) for 60 min at 4 °C. The precleaned lysates were treated with rabbit anti-p62 antibody or normal rabbit IgG. Immune complexes were precipitated by protein G-sepharose beads for 2 h at 4 °C. The beads were then washed and boiled in SDS lysis buffer, and the proteins released from the beads were subjected to Western blotting.

### ROS detection assay

Cells (1.5 × 10^4^) were seeded in 96-well glass plates (Matsunami Glass Ind., Ltd). The next day, the cells were transfected with siRNA using Lipofectamine RNAiMAX. At 48 h after transfection, the cells were treated with 5 μM CM-H2DCFDA (General Oxidative Stress Indicator; C6827; Thermo Fisher Scientific) for 30 min in PBS treated with 0.5 mM MgCl2, 0.9 mM CaCl2, and 0.4% FBS. The cells were then treated with dopamine with complete medium for 6 h. The mean fluorescence intensity (MFI) of CM-H2DCFDA was analyzed by fluorescence microscopy, and the amount of ROS in the cells was measured by subtracting the MFI of noncellular regions from the MFI of cells using the FIJI ImageJ software program. Cellular and noncellular regions were distinguished by bright field microscopy. All experiments were performed in a phenol red-free medium.

### Cell viability assay

Cell viability was measured using a Cell Counting Kit-8 (CCK-8) (DOJINDO). SH-SY5Y cells were cultured on 96-well plates for 24 h. Cells were transfected with siRNA (USP10-siRNA, p62-siRNA, Nrf2-siRNA, Keap1-siRNA, or the control siRNA) using Lipofectamine RNAiMAX. At 48 h after transfection, cells were treated with 0.1 to 0.4 mM dopamine for 12 h and then with the CCK-8 kit containing WST-8 in fresh medium for 1 h, and the absorbance (485 nm) of culture medium was measured using a TriStar LB 941 Microplate Reader (Berthold).

### Quantification of mRNA

Total RNA was extracted from SH-SY5Y cells using an RNA extraction kit (NucleoSpin RNA; TAKARA) according to the manufacturer’s protocol. Total RNA (5 ng) was subjected to reverse transcription and polymerase chain reaction (RT-PCR) reaction using a One Step TB Green PrimeScript RT-PCR Kit II (TAKARA) and a primer set. Real-time RT-PCR was performed using a Thermal Cycler Dice Real Time System (TAKARA). The NQO1, GSTM1, p62, and Nrf2 mRNA levels were normalized according to the β-actin mRNA level. The sequences of the primers used for real time RT-PCR were as follows: NQO1 forward is ccctgcgaactttcagtatcc, and reverse is ctttcagaatggcagggactc; GSTM1 forward is tgcccatgatactggggta, and reverse is gccactggcttctgtcataat; p62 forward is cacctgtctgagggcttctc, and reverse is cacactctccccaacgttct; Nrf2 forward is cggtatgcaacaggacattg, and reverse is agaggatgctgctgaaggaa, β-actin forward is gacaggatgcagaaggagatcac, and reverse is gtcatactcctgcttgctgatcc.

### Statistical analyses

Data were analyzed with a one-way analysis of variance (one-way ANOVA) followed by Tukey's multiple comparisons test using the Prism8 software program (GraphPad). The data in Figures 1*D* and 12*C* did not meet the requirement of a one-way ANOVA that the standard deviations of all samples are comparable, as assessed by the Brown–Forsythe test. Therefore, the significances of the difference in Figures 1*D* and 12*C* were calculated by Brown–Forsythe and Welch ANOVA followed by Dunnett's T3 multiple comparisons test.

## Data availability

All data are contained within the manuscript.

## Conflict of interest

The authors declare no conflicts of interest in association with the present study.
